# Design and experiment of miss-seeding detection and preparatory seed scraper-belt compensation mechanism based on improved YOLOv5s for potato seed-metering devices

**DOI:** 10.3389/fpls.2025.1686174

**Published:** 2025-10-16

**Authors:** Hongling Li, Hua Zhang, Xiaolong Liu, Hui Li, Shangyun Jia, Wei Sun, Guanping Wang, Quan Feng, Sen Yang

**Affiliations:** College of Mechanical and Electrical Engineering, Gansu Agricultural University, Lanzhou, China

**Keywords:** potato, spoon-chain seed-metering device, visual detection, automatic reseeding, improved YOLOv5s

## Abstract

This study addresses the issue of miss seeding in spoon-chain potato seed-metering devices, which impacts planting efficiency and quality, by proposing a miss-seeding detection and compensation system based on an improved YOLOv5s model integrated with a preparatory seed scraper-belt compensation mechanism. The enhanced model incorporates the Convolutional Block Attention Module (CBAM) and Soft Non-Maximum Suppression (Soft-NMS), achieving a mean average precision (*mAP*) of 99.40% in complex field environments. The system combines visual recognition with mechanical compensation. Experimental results demonstrate that at operating speeds of 0.2–0.4 m/s, the original miss-seeding rate of 5.28%–9.40% is reduced to 0.70%–1.68%, with a reseeding success rate of 82.14%–86.67% and a preparatory seed reseeding success rate exceeding 96%. The study validates the system’s efficiency and reliability under medium-low speeds, with slight performance degradation at higher speeds due to vibrations. This solution offers an intelligent upgrade path for traditional potato seed-metering devices and advances precision agriculture technologies.

## 1 Introduction

The potato is a crucial food crop globally, and the level of mechanized planting significantly impacts its yield and profitability. The spoon-chain seed metering device is commonly employed in potato planter due to its uncomplicated design and versatility. Nevertheless, challenges arise from the inherent physical attributes of seeds, such as uneven size and surface viscosity, as well as operational factors like vibration and dust. Consequently, miss-seeding occurrences are common, resulting in uneven emergence and reduced yields in the field (Wang et al., 2020). Hence, the exploration of effective detection and compensation technologies for miss-seeding is essential to enhance the efficiency of mechanized potato planting.

Traditional miss-seeding detection methods predominantly rely on photoelectric sensors, ultrasonic sensors, or mechanical contact detection. For instance, Zhang et al. (2013) designed a miss-seeding detection system based on infrared photoelectric sensors combined with a stepper motor for reseeding. However, this approach is susceptible to interference from field dust during operation. Wen et al. (2022) proposed a miss-seeding detection and reseeding system for belt-spoon type seed-metering devices, utilizing photoelectric sensors and electromagnetic actuators. It achieved a 100% detection success rate and 83% reseeding success rate within a speed range of 0.14–0.54 m/s. While the system benefits from rapid response, its adaptability to complex field environments remains limited. Additionally, Guan et al. (2021) explored multi-sensor fusion for miss-seeding detection, which improved robustness but increased system complexity and cost.

To address the insufficient anti-interference capability of photoelectric sensors, recent studies have shifted toward alternative detection techniques. Wang et al. (2023) developed a miss-seeding detection method based on spatial capacitive sensors, employing the AD7745 high-precision capacitive chip to determine miss-seeding status by detecting net changes in capacitance values as seed spoons pass through capacitive electrode plates. Lei et al. (2022) designed a miss-seeding detection and reseeding system using a “displacement positioning method,” integrating permanent magnet arrays, Hall sensors, and diffuse-reflective photoelectric switches, achieving a detection accuracy of 96.54%. While these methods perform well under specific conditions, they exhibit limitations in high-speed operations or dynamically variable environments.

In recent years, the application of deep learning in agricultural machinery has introduced novel technical pathways for miss-seeding detection (Rizvi et al., 2024; Thomas et al., 2021). Object detection algorithms based on Convolutional Neural Networks (CNNs) have been widely adopted in agricultural scenarios due to their efficiency and accuracy. For example, Faster R-CNN, utilizing a Region Proposal Network (RPN), achieves high precision in crop disease detection. Liang et al. (2024) applied this algorithm to identify corn leaf disease spots, attaining a mean average precision (*mAP*) of 92.8%. At the same time, numerous new technologies have emerged in object detection. Joint detection and tracking methods based on reinforcement learning have demonstrated substantial potential in radar target recognition (Li et al., 2024b). Additionally, significant breakthroughs in hyperspectral image analysis, open set recognition, and underwater color difference studies have provided crucial insights for visual perception in complex environments (Xu et al., 2025a, Xu et al., 2025b; Li et al., 2025). These technological advancements offer new possibilities for enhancing the performance of agricultural visual detection tasks.

In agricultural applications with high real-time requirements, YOLO series algorithms are highly favored due to their outstanding speed advantage. Kang et al. (2020); Deng et al. (2025) developed a vision system for fruit-picking robots based on YOLOv3, achieving a detection speed of 25 frames per second (FPS) and an 87% recognition rate in complex orchard environments. As the latest iteration, YOLOv5 further optimizes network architecture and computational efficiency (Sun et al., 2025). Its lightweight design makes it particularly suitable for deployment on embedded platforms (e.g., NVIDIA Jetson series) and widely used in fields such as autonomous agricultural machinery and crop growth monitoring (Jocher et al., 2020, Nnadozie et al., 2023). Despite the YOLO series advancing to YOLOv13, YOLOv5s retains notable advantages, particularly on resource-constrained embedded hardware platforms (Lawal et al., 2023; Li et al, 2024b). Compared to YOLOv13, YOLOv5s significantly reduces computational requirements, which make it more suitable for efficient real-time detection on low-power devices (Lazarevich et al., 2023). In the agricultural sector, especially in mechanized planting, there is a need for deploying systems on low-cost, high-performance compact devices. The optimized YOLOv5s model structure enhances real-time processing and reduces computational costs, effectively meeting the detection demands of agricultural machinery in dynamic environments. It is particularly well-suited for embedded systems (e.g., NVIDIA Jetson TX2) and can perform real-time miss-seeding detection tasks, without adversely affecting the device’s power consumption or performance (Wu et al., 2023).

However, the original YOLOv5 still faces limitations in complex field environments. Due to background noise (e.g., weeds, soil texture, lighting variations) and target diversity (e.g., visual similarity between seeds and soil), the model is prone to missed or false detections (Tan et al., 2025), leading to reduced accuracy (Xu et al., 2024; Liu et al., 2025). To address this, researchers have proposed various improvements. The integration of attention mechanisms significantly enhances the model’s ability to focus on critical features. Woo et al. (2018) proposed the Convolutional Block Attention Module (CBAM), which strengthens the representational capacity of target regions through spatial and channel attention, improving *mAP* by approximately 2%–3% in object detection tasks. Bodla et al. (2017) validated its effectiveness on the COCO dataset. These enhancements broaden YOLOv5’s applicability in complex agricultural scenarios.

Notably, visual-based research on miss-seeding detection for potato seed-metering devices remains scarce. Existing technologies predominantly focus on traditional sensor-based methods (Wang et al., 2024; Zhang et al., 2022; Qiu et al., 2023), which struggle to meet the demand for efficient detection under complex field conditions. This gap underscores the vast potential for deep learning in advancing potato miss-seeding detection technologies.

In summary, potato seed-metering devices still face challenges in miss-seeding detection and compensation technology:

In response to the above challenges, this study proposes a miss-seeding detection and preparatory seed scraper-belt compensation mechanism based on improved YOLOv5s model for a spoon-chain potato seed-metering device, At the software level, integrating the CBAM attention mechanism and the Soft-NMS optimization algorithm enhances the model’s feature extraction capability in dusty, complex backgrounds, and overlapping seed conditions, significantly improving the accuracy and robustness of miss-seeding detection. At the hardware level, the innovative scraper-belt compensation mechanism, which involves precise seed presetting and rapid reseeding, substantially enhances stability and efficiency during high-speed operations. The collaboration between the NVIDIA Jetson TX2 (for visual inference) and the STM32 microcontroller (for mechanical control) creates a ‘perception-decision-execution’ closed-loop control system. This integration effectively addresses the disconnection between detection and reseeding phases in existing methods, resulting in efficient miss-seeding detection and accurate compensation in complex operational environments.

## 2 Overall design

### 2.1 The basic structure of the system

**Figure 1:**
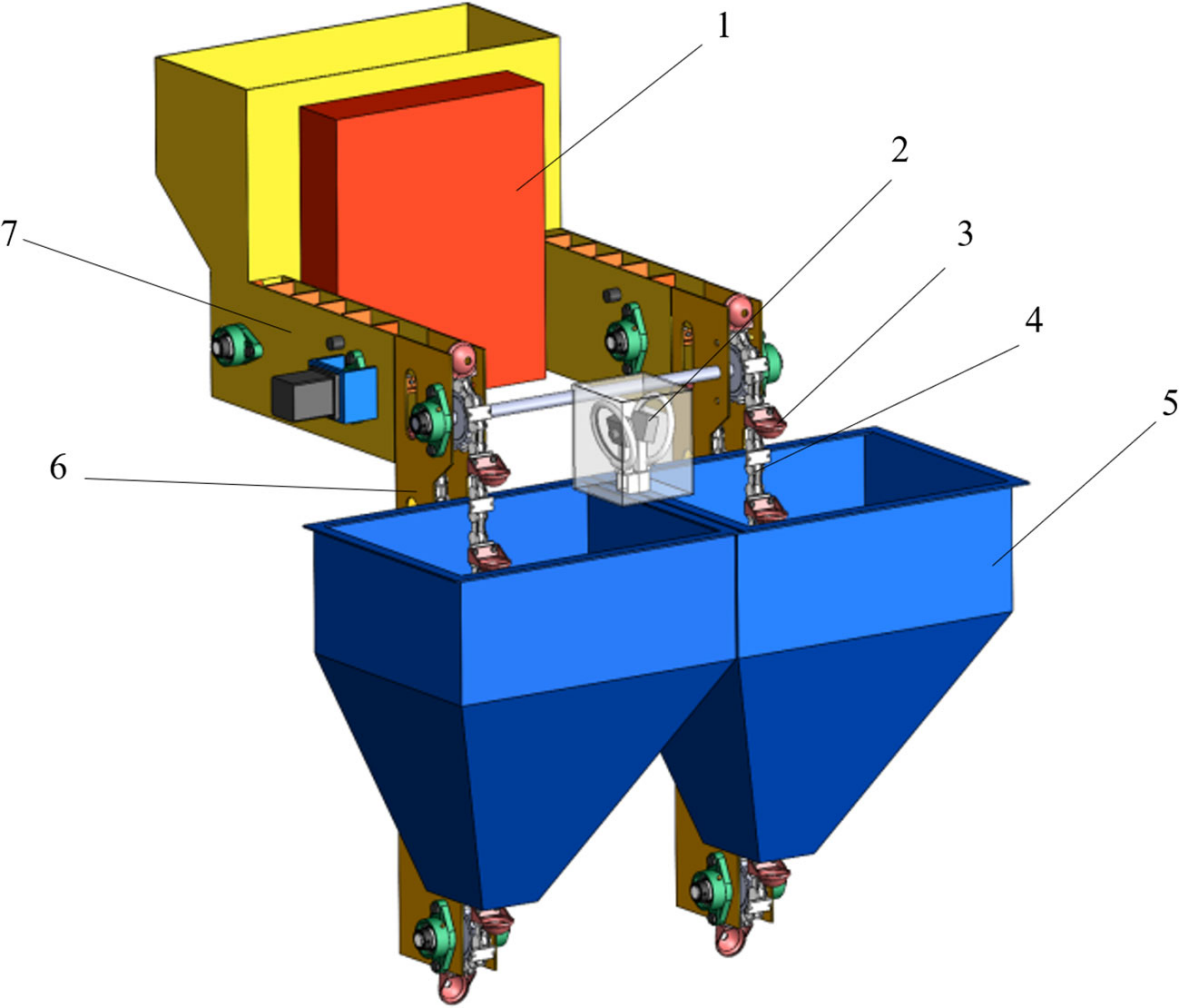
Structural scheme diagram of the miss-seeding detection and compensation system of spoon-chain metering device. 1. Storage box; 2. Miss-seeding detection device; 3. Seed spoon; 4. Seed-metering chain; 5. Seed box; 6. Seed protection groove; 7. Reseeding device.

The spoon-chain seed-metering device primarily consists of a seed box, seed spoons, seed-protecting grooves, and a seed-metering chain. As shown in **Figure 1**, each device is equipped with a miss-seeding detection unit and a preparatory seed scraper-belt compensation mechanism unit.When a potato seed is lifted to its highest point using a seed spoon, it naturally falls back down due to gravity onto the backside of the previous spoon and a compensation port is located at this natural drop point. A reseeding belt with L-type scrapers is installed on the seed-protecting groove near the compensation port. A fiber optic sensor is mounted at the front end of the reseeding belt to monitor seed availability. A reseeding box is installed above the end of the reseeding belt, featuring a seed outlet at its bottom. To prevent potato seeds from jamming, the outlet size is designed to accommodate a single potato seed. The reseeding box also houses a storage box containing the NVIDIA Jetson TX2 (for model deployment), a microcontroller, motor driver, and an LCD display.

1.Storage box 2.Miss-seeding detection device 3.Seed spoon 4.Seed-metering chain 5.Seed box 6.Seed protection groove 7. Reseeding device.

### 2.2 Hardware composition

**Figure 2:**
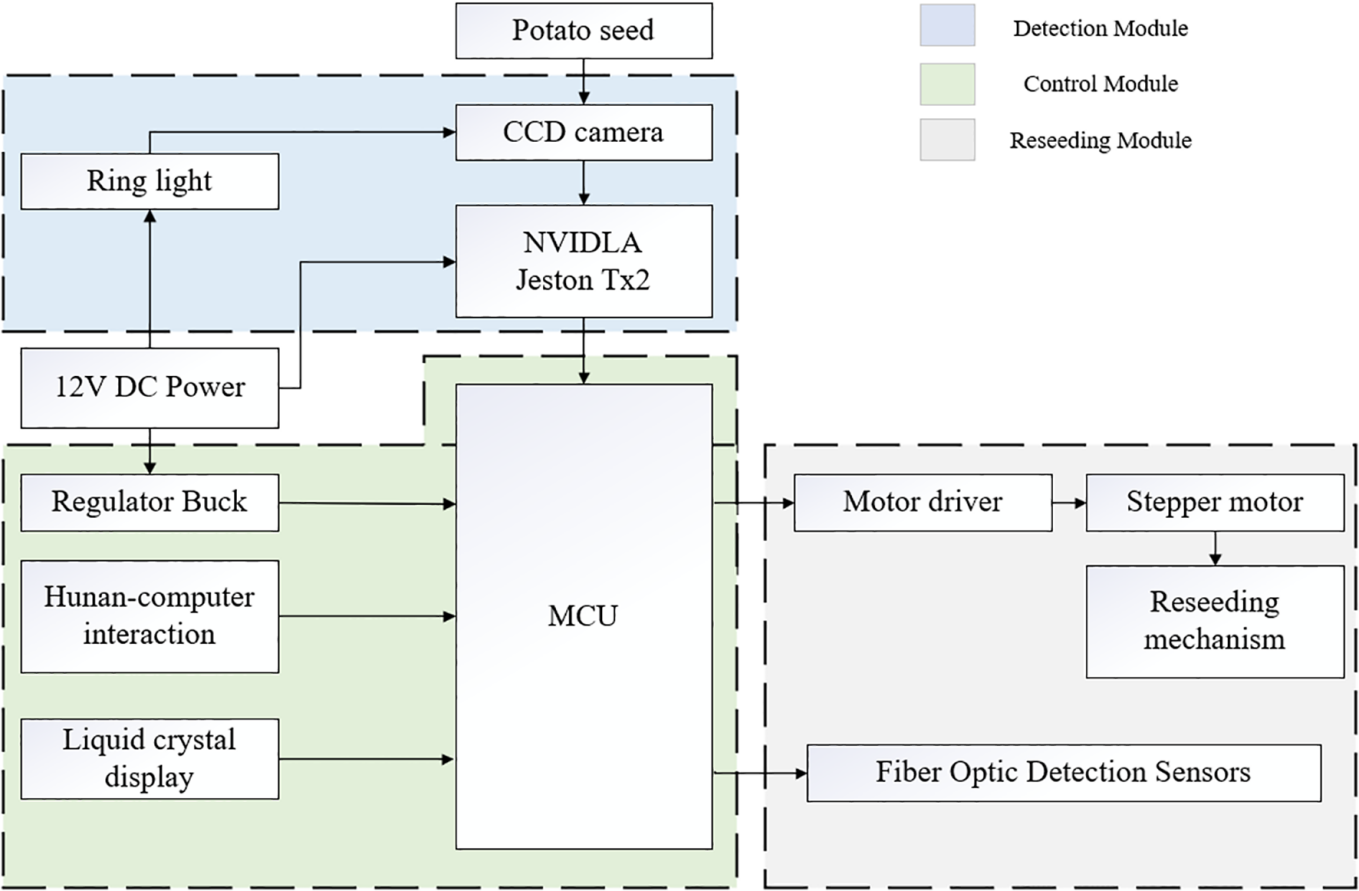
The hardware structure diagram of the system. The hardware structure includes three modules with Detection, Control, and Reseeding. Detection uses CCD camera, NVIDIA Jetson TX2 and ring light; Control involves MCU, human-computer interaction, and liquid crystal display, Reseeding employs motor driver, stepper motor, fiber optic sensors and reseeding mechanism.

The hardware structure of the miss-seeding detection and reseeding system is shown in **Figure 2**. The hardware components of the miss-seeding and compensation system mainly include a miss-seeding detection module, a reseeding module, and a control module. Among these, the miss-seeding detection module includes an embedded AI computing device (NVIDIA Jetson TX2), a CCD industrial camera, and ring light; the reseeding module comprises a fiber optic sensor, driver, stepper motor, and reseeding mechanism (reseeding belt and reseeding box). The STM32F103ZET6 microcontroller is selected as the lower-level controller, which controls the reseeding device to prepare and replenish seeds based on detection results from the fiber optic sensor and the upper-level controller NVIDIA Jetson TX2. The system is powered by a 12V DC vehicle power supply from the tractor.

### 2.3 Working principle

The spoon-chain seed-metering device moves upward with the implement during operation. When a seed spoon enters the field of view of the CCD industrial camera, the camera captures real-time video of the spoon’s motion. The video stream is transmitted to the NVIDIA Jetson TX2 development board via a USB 3.0 interface. The Jetson TX2, running an improved YOLOv5s miss-seeding detection algorithm, analyzes whether the seed spoon contains a potato seed. If a seed is detected, the system proceeds with normal seeding. If no seed is detected (empty spoon), the Jetson TX2 sends a signal to the STM32 microcontroller which generates a reseeding pulse. This pulse drives the stepper motor through a driver, activating the reseeding mechanism to replenish the missed seed.

Prior to reseeding, the system prepares seeds through the following process: The distance between two L-type scrapers on the reseeding belt defines a working interval. Upon system startup, the stepper motor drives the reseeding belt to move. The L-type scrapers push potato seeds from the seed outlet toward the seed-metering device. When a working interval reaches the fiber optic sensor’s detection position, the sensor checks for seed presence by monitoring light path obstruction. If no seed is detected, the stepper motor advances the belt to the next interval. The process continues until a seed is detected within the working interval, at this point, the motor stops, completing seed preparation and waiting for reseeding signals.

## 3 Design of miss-seeding detection and reseeding system

This system adopts a serial workflow of “visual detection first, followed by mechanical compensation,” which is determined based on a comprehensive consideration of system reliability, timing synchronization, and implementation complexity. The fundamental principle of this method is that miss seeding depends on accurate identification through visual detection. The detection process inherently includes delays in image capture, model analysis, and data transmission. Additionally, the mechanical reseeding system needs clear instructions and a strict time frame to perform its tasks. The serial design ensures that compensation is triggered only after the detection results are reliably confirmed, thus preventing ineffective compensation or misactions due to data uncertainty or immature timing. In contrast, while parallel or reverse designs may theoretically reduce response time, they increase the risk of misactions in high-speed agricultural operations, resulting in seed wastage or inaccurate compensation. Therefore, the serial workflow chosen in this study balances reliability, accuracy, and feasibility under current technological conditions.

### 3.1 Miss-seeding detection module

**Figure 3:**
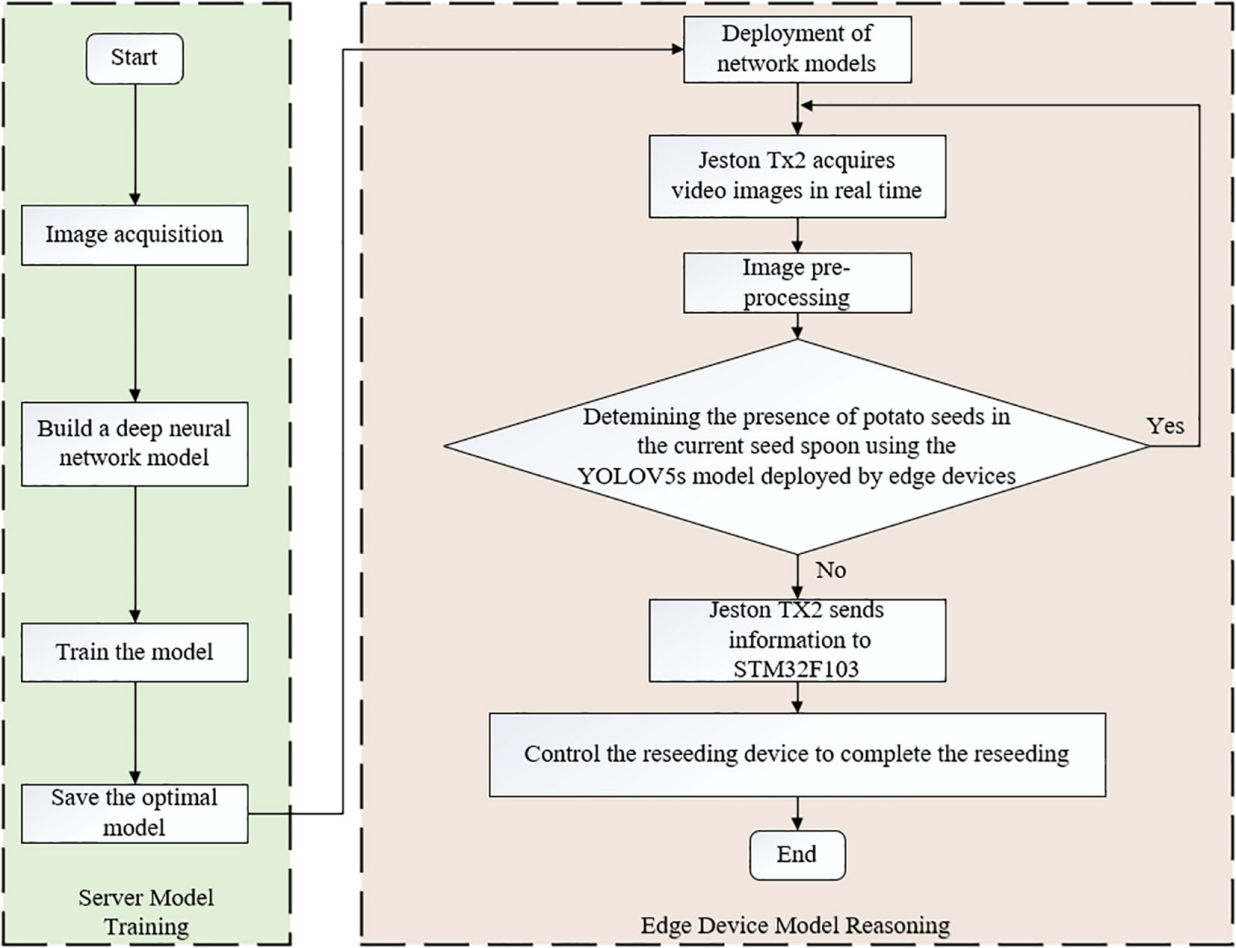
Miss-seeding detection control flowchart. This diagram presents the closed-loop process in chronological order, including data collection and annotation, server-side training and model conversion, edge-side inference and instruction issuance, and the execution of supplementary broadcasting. After the detection module outputs the target box and category, the control module determines whether supplementary broadcasting is required based on the particle’s state and displacement information, and sends action instructions to the stepper motor driver.

The operation of the detection system consists of two stages: server training and model deployment, as shown in **Figure 3**. Firstly, a dataset of seed spoons is collected and annotated under various scenarios, followed by offline model training using the custom dataset on the server. Subsequently, the NVIDIA Jetson TX2 development board, specifically designed for visual applications and machine learning tasks, is selected to deploy the neural network model. The converted model is optimized using NVIDIA TensorRT to enhance inference performance. During operation, the spoon-chain seed-metering device moves upward sequentially with the implement. When a seed spoon reaches the monitoring camera module, the camera captures real-time motion video of the seed-metering device. The YOLOv5s object detection algorithm deployed on the Jetson TX2 is then utilized to detect potato seeds. By integrating the RS-485 interface and Modbus RTU protocol, commands are sent to the lower-level STM32 microcontroller to obtain information on whether the current spoon has missed seeding. If a potato seed is detected in the spoon, the model continues monitoring. Otherwise, upon receiving the miss-seeding data, the microcontroller executes a program to send a reseeding command to the compensation system, completing the reseeding process.

**Figure 4:**
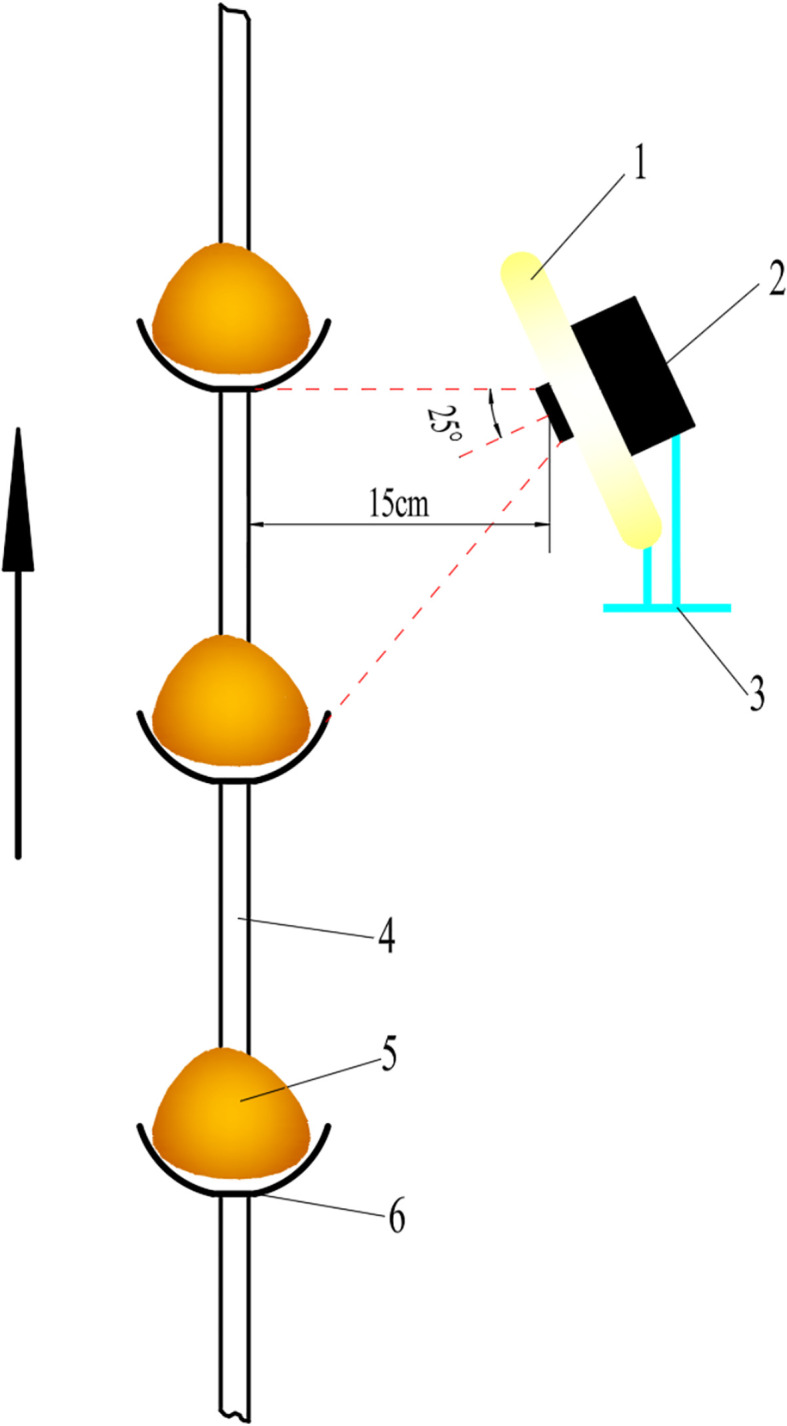
Structural diagram of the miss-seeding detection module. 1. Ring light; 2. CCD camera; 3. Aluminum alloy bracket; 4. Seed-metering chain; 5. Potato seed; 6. Seed spoon.

The structural schematic of the miss-seeding detection module is shown in **Figure 4**. The CCD industrial camera is mounted at the front end of an aluminum alloy bracket above the seed box, with its lens angled downward at 25 to align with the motion trajectory of the seed spoons on the seed-metering chain, enabling real-time detection of whether the upward-moving spoons are properly loaded with potato seeds. The center of the camera lens maintains a horizontal distance of 15 cm from the seed-metering chain with a field of view covering a detection area of approximately 10 cm × 8 cm. An LED ring light is installed at the same horizontal level as the camera to provide supplemental illumination. Both camera and ring light unit are enclosed in an IP65-rated transparent dustproof enclosure. The bracket is fixed with vibration-damping pads to ensure stability under demanding operating conditions.

### 3.2 Reseeding module

**Figure 5:**
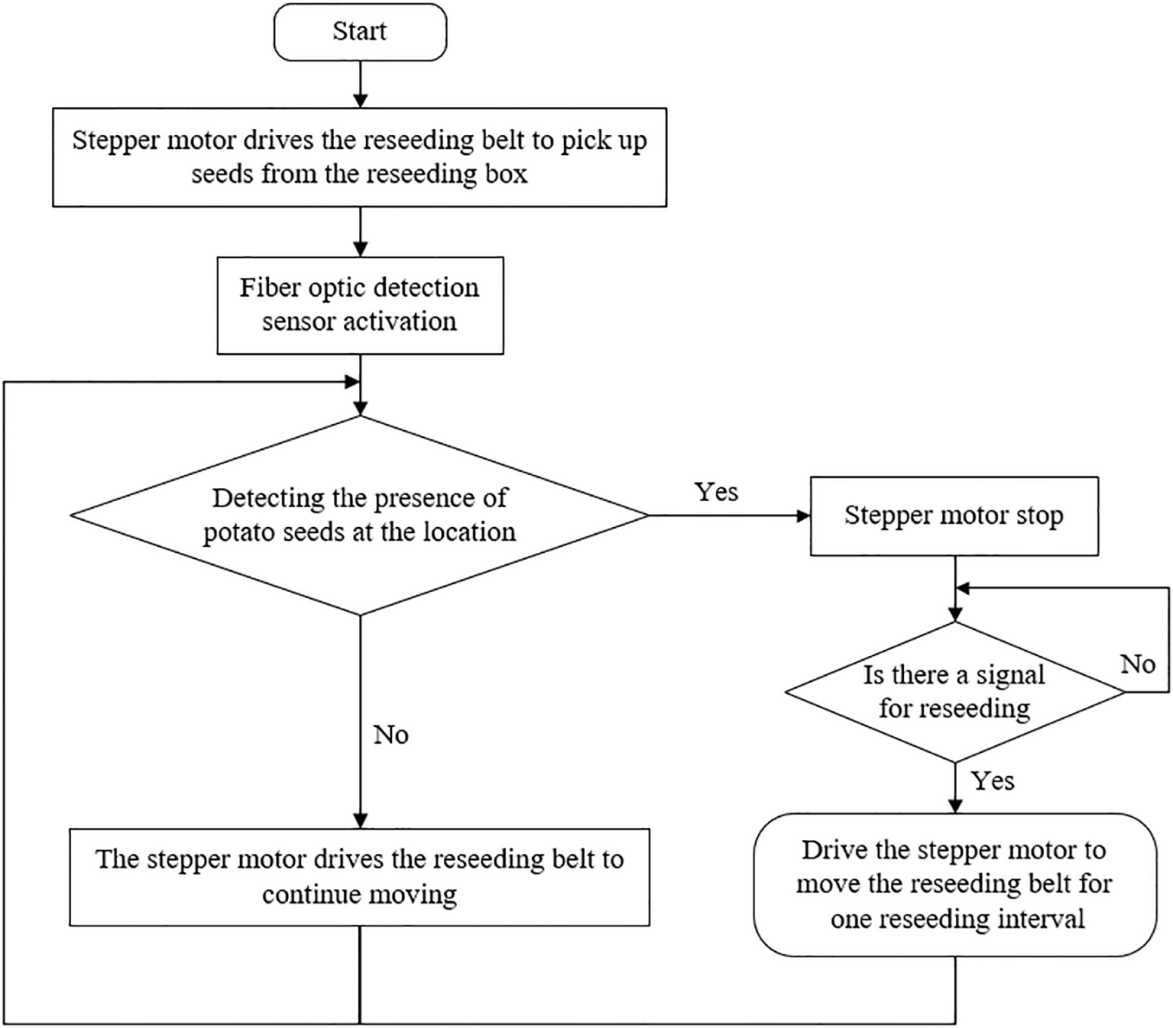
Flowchart illustrating a reseeding process using a stepper motor and fiber optic detection sensor. The process starts with the motor driving the reseeding belt to pick up seeds. After activation of the sensor, the system checks for the presence of potato seeds. If detected, the motor stops.If not, the motor continues moving. Upon stopping, a check for a reseeding signal occurs. If there is a signal, the motor advances the belt for one reseeding interval. If no signal, the process repeats.

The control flowchart of reseeding system is illustrated in **Figure 5**, encompassing seed preparation and reseeding processes. Upon system power-up, the microcontroller sends a signal to activate the stepper motor via the driver, driving the reseeding belt into motion while simultaneously initiating the fiber optic sensor. The system determines whether seeds are present on the reseeding belt at the detection position based on changes in the sensor signal. If no seeds are detected by the fiber optic sensor, the stepper motor continues moving the reseeding belt; otherwise, the motor halts, completing seed preparation and awaiting the microcontroller’s reseeding command. Once the microcontroller issues a reseeding pulse, the stepper motor drives the reseeding belt to advance by one working interval, completing the reseeding action before cycling to the next operation.

**Figure 6:**
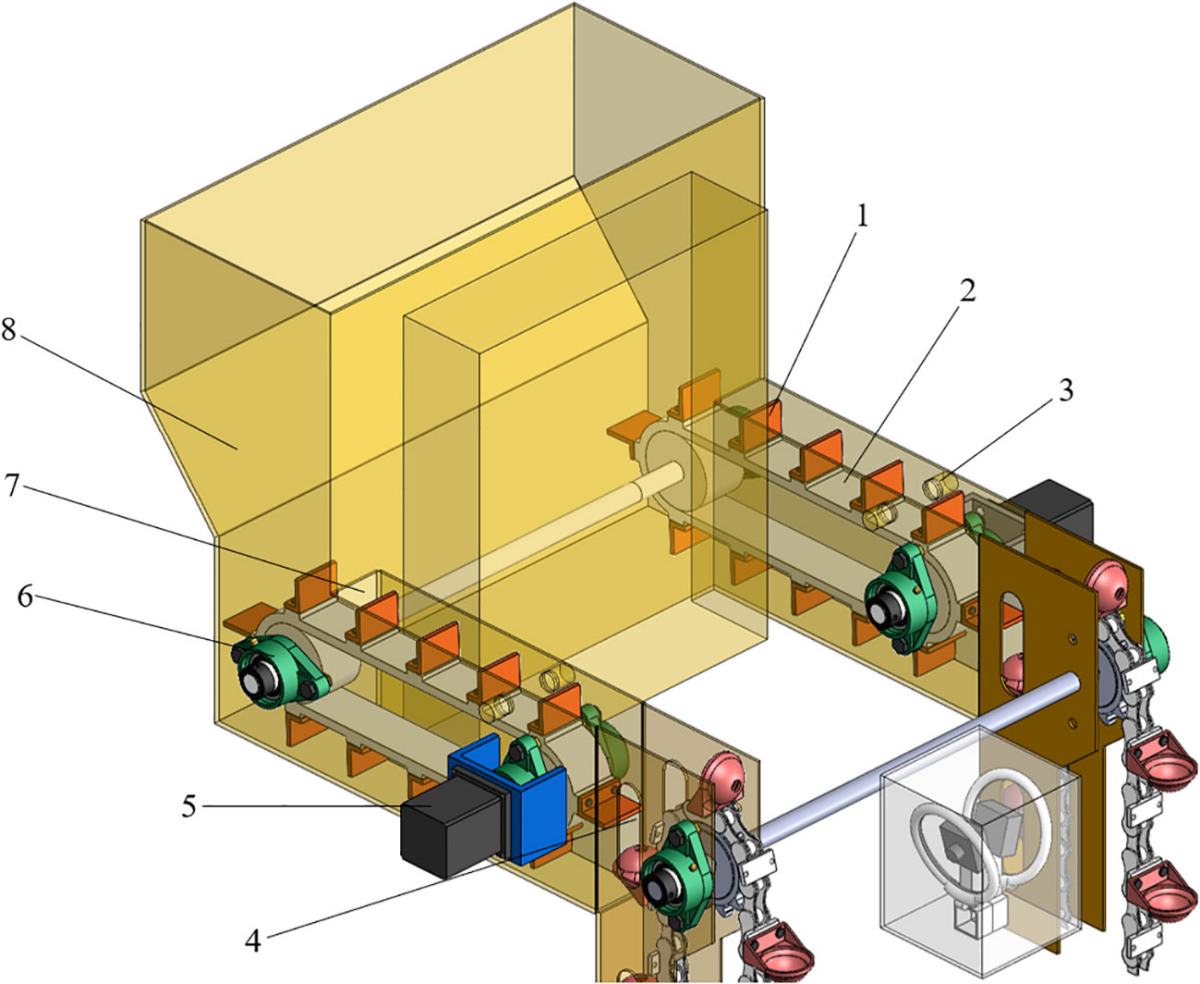
Structural diagram of the reseeding device. 1. L-type scraper; 2. Reseeding belt; 3. Fiber optic sensor; 4. Seed compensation port; 5. Stepper motor; 6. Reseeding follower wheel; 7. Seed outlet; 8. Reseeding box.

The reseeding device, as shown in **Figure 6**, primarily comprises a fiber optic sensor, driver, stepper motor, reseeding box, reseeding belt with L-type scrapers, and seed-protecting groove. The stepper motor driver is directly powered by the tractor’s 12V DC vehicle power.

### 3.3 Control circuit

**Figure 7:**
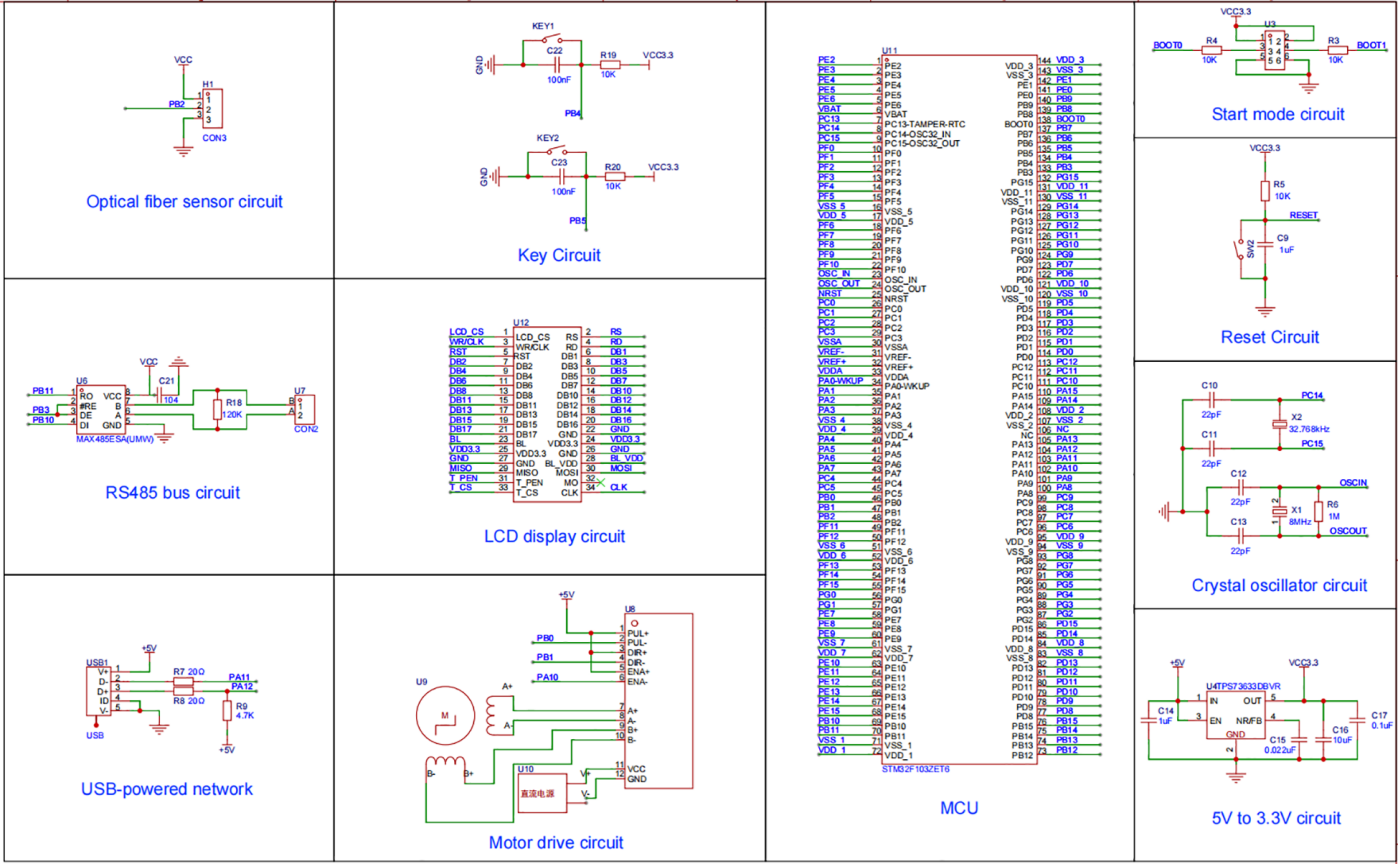
Diagrams of electronic circuits. Diagrams of electronic circuits for various modules including optical fiber sensor, key circuit, RS485 bus, LCD display, start mode, reset, crystal oscillator, USB-powered network, motor drive, and 5V to 3.3V. The central section displays a detailed microcontroller unit (MCU) pinout. Each diagram includes labeled components and connections.

The control circuit, as shown in **Figure 7**, centers on the STM32F103ZET6 microcontroller, selected for its Cortex-M3 core, 72 MHz clock speed, and rich peripheral resources (e.g., UART, PWM, GPIO), which meet real-time control requirements. The fiber optic detection circuit employs a through-beam fiber optic sensor (MEIJIDENKI, PD-62) connected to the microcontroller via the PB2 port to monitor the seed preparation status of the reseeding belt. The driver circuit, paired with the HY42DJ60 stepper motor, uses a TB6600 driver, with PWM signals from the PB0 port controlling motor speed and the PB1 port setting motor direction. Two buttons connected to PB4 and PB5 enable manual start/stop and mode switching. The liquid crystal display utilizes a 3.5-inch TFT-LCD module supplied by ALIENTEK to provide real-time feedback on miss-seeding count, reseeding success rate, and system status. The microcontroller’s UART interface communicates with the upper-level NVIDIA Jetson TX2 via differential signal conversion using a MAX485 chip, operating at a baud rate of 9600 bps and supporting remote monitoring via the Modbus protocol. An LDO voltage regulator steps down the 5V power supply to 3.3V for the microcontroller. All modules are integrated via a PCB, with shielded cables ensuring stable communication and coordinated control in complex field environments.

## 4 Design of miss-seeding detection algorithms

### 4.1 Potato seed image acquisition

This experiment was conducted at the Potato Cultivation Base of Gansu Agricultural University during 2023–2024. A high-resolution CCD industrial camera (Model: WP-UT320/M, frame rate: 120 FPS) was used to capture images of potato seeds, with an image resolution of 2048×1536. Static images were obtained by extracting frames from videos of moving seed spoons, and high-quality samples were selected through data filtering to construct the image dataset. Image annotation, a critical step for model training, employed a manual labeling method, where “Potato seed” and “Miss seeding” tags were assigned to indicate the presence of potato seeds and miss-seeding status, respectively. A total of 5,000 annotated images were generated. The final dataset was divided into training, validation, and test sets in an 8:1:1 ratio. Specifically, the training set comprises 4,000 images, the validation set contains 1,000 images, and the test set includes 1,000 images, with each subset meeting specific requirements for data quality and quantity.

### 4.2 YOLOv5s algorithm overview

**Figure 8:**
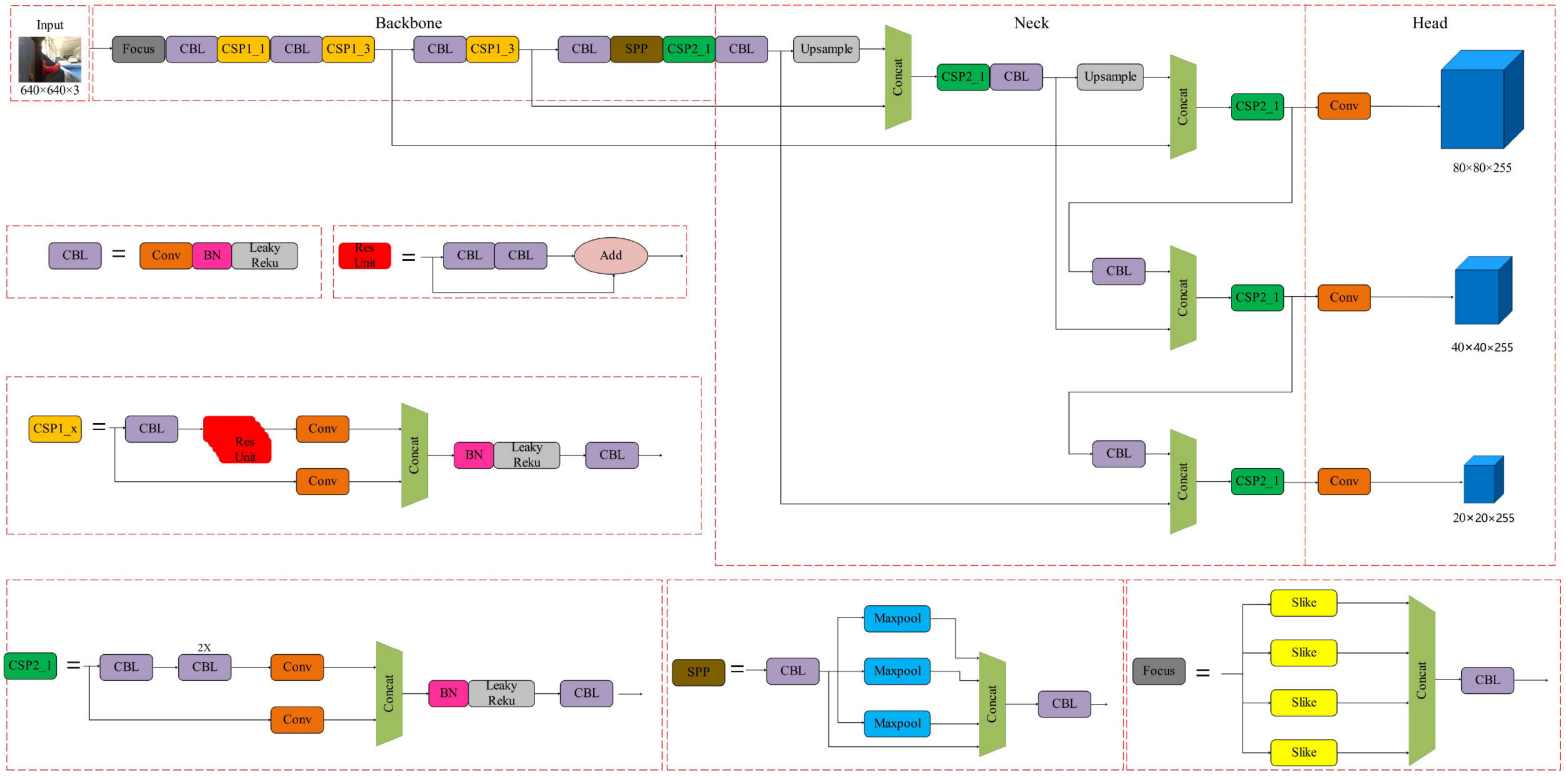
Diagram of YOLOv5s network architecture. The network architecture consisting of input, backbone, neck, and head stages. The backbone includes Focus, CBL, CSP modules, and an SPP block, progressing through upsample layers. The neck features CSP2 modules connected by concatenation. The head comprises convolution layers outputting three distinct dimensions: 80×80×255, 40×40×255, and 20×20×255. Detailed sections show the internal structure of CBL, Res Unit, CSP modules, and SPP block.

To meet the operational requirements of agricultural machinery, this study employs the YOLOv5s algorithm, known for its higher accuracy, stronger generalization, and lightweight design. The network architecture, illustrated in **Figure 8**, comprises four main components: Input, Backbone, Neck, and Head (Li et al., 2024a). After preprocessing the seed spoon images to match the model’s input dimensions, forward propagation is performed through the backbone network. YOLOv5s utilizes CSPDarknet53 as its backbone to extract image features. The Neck module integrates an FPN+PAN structure, where the Feature Pyramid Network (FPN) propagates semantic information top-down, and the Path Aggregation Network (PAN) transmits localization information bottom-up, generating multi-scale feature maps for the Head module. The Head module, designed for lightweight efficiency, combines multi-scale convolutions and upsampling operations to output predictions for each grid cell, including a fixed number of bounding boxes and their class confidence scores. To reduce redundant detections, the Non-Maximum Suppression (NMS) algorithm is applied to retain the highest-confidence bounding boxes while suppressing others with Intersection over Union (IoU) exceeding a preset threshold (Symeonidis et al., 2023).

The objective function during the training phase consists of three components: boundary box regression, object confidence, and class classification. Let the *i*-th anchor and its corresponding ground truth box be $b_{i}$ and $b_{i}^{*}$ , respectively. Let ${\hat{p}}_{i}$ represent the predicted probability of object presence, $y_{i}$ ∈{0,1} be the indicator variable, and ${\hat{q}}_{i}$ be the predicted class distribution. The total loss is given by Equation 1. Here, *BCE*(*a*,*b*) represents the binary cross-entropy loss, as shown in Equation 2. The boundary box regression is based on the Complete Intersection over Union (CIoU), as described in Equations 3–5. In these equations, ( x , y , w , h ) and ( $x^{*}$ , $y^{*}$ , $w^{*}$ , ${\text{h}}^{*}$ ) denote the center and scale of the predicted and ground truth boxes, respectively, ρ is the Euclidean distance between the center points, and c is the diagonal length of the smallest enclosing box. $\lambda_{box}$ , $\;\lambda_{obj}$ , $\lambda_{cls}$ are the loss weights. To align with the baseline, the training strategy and weight settings remain consistent.


(1) $$\begin{array}{c} ℒ=\lambda_{box}\sum_{i}{ℒ}_{C}IoU\left(b_{i},b_{i}^{*}\right)+\lambda_{obj}\sum_{i}BCE\left({\hat{p}}_{i},y_{i}\right)+\lambda_{cls}\sum (i:y_{i}\\ =1)BCE\left({\hat{q}}_{i},q_{i}^{*}\right) \end{array}$$

(2) BCE(a,b)=−[b ln(a) + (1−b) ln(1−a)]

(3) $${ℒ}_{C}IoU\left(b,b^{*}\right)=1-IoU\left(b,b^{*}\right)+\rho^{2}\left(\left(x,y\right),\left(x^{*},y^{*}\right)\right)/c^{2}+\alpha v$$

(4) $$v=4/\pi^{2}{\left(arctan\left(w^{*}/h^{*}\right)-arctan\left(w/\text{h}\right)\right)}^{2}$$

(5) $$\alpha =v/\left(1-IoU\left(b,b^{*}\right)+v\right)$$

### 4.3 Algorithmic improvements

#### 4.3.1 Introduce the CBAM attention mechanism

**Figure 9:**
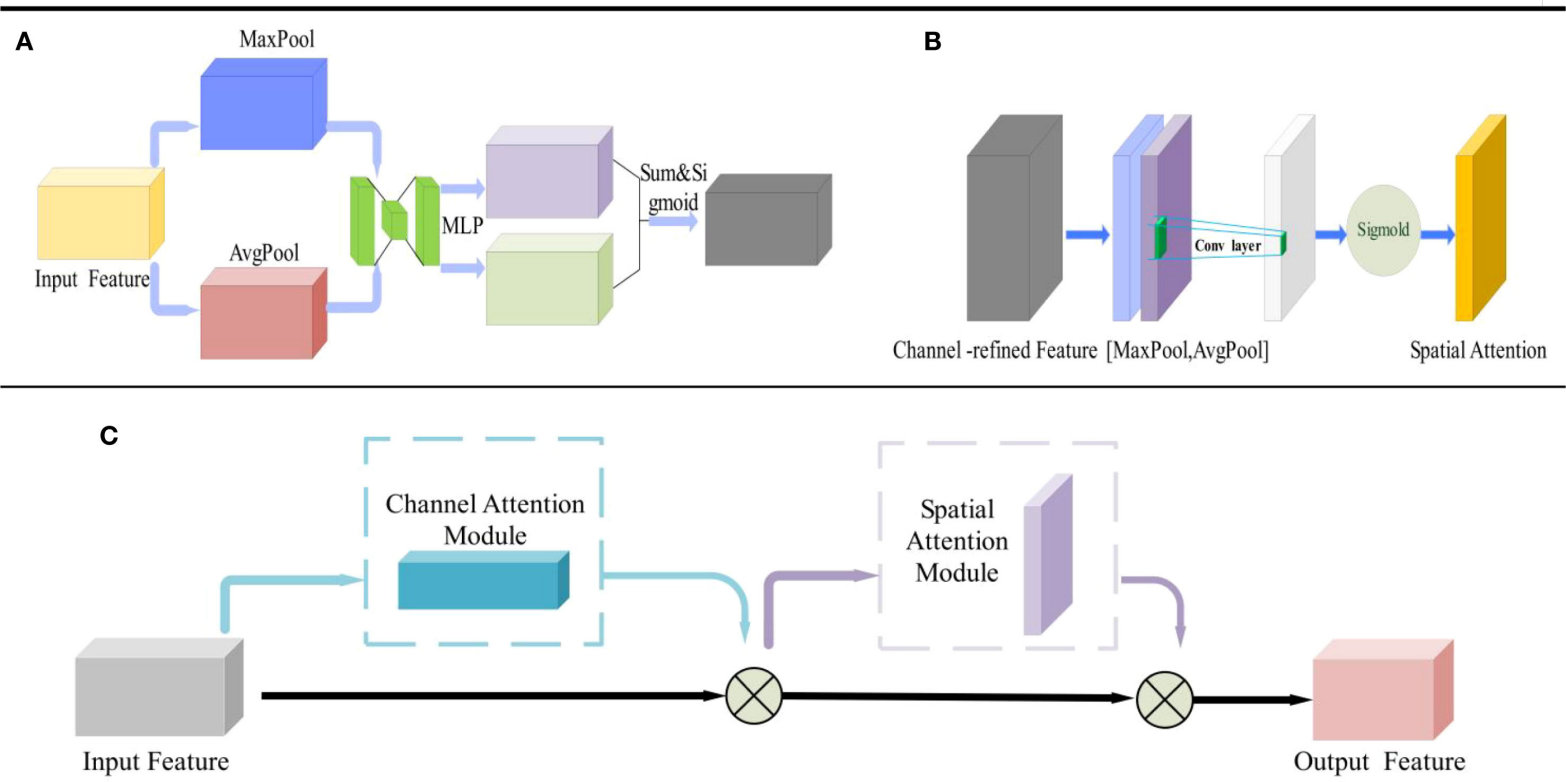
Structural diagram of CBAM. **(A)** Channel attention module; **(B)** Spatial attention module; **(C)** Convolutional block attention module.

To mitigate the impact of complex field backgrounds and high-dust environments on potato miss-seeding detection performance during agricultural operations and enhance the model’s adaptability to challenging scenarios, this study integrates the Convolutional Block Attention Module (CBAM) into the original YOLOv5s framework, as illustrated in **Figure 9**. The CBAM comprises two submodules: the Channel Attention Module (CAM, **Figure 9A**) and the Spatial Attention Module (SAM, **Figure 9B**). The CAM enhances feature representation of critical channels by applying attention weights to each channel of the feature map, while the SAM assigns spatial attention weights to prioritize key spatial regions. The combined CBAM (**Figure 9C**) dynamically learns both channel-wise and spatial attention distributions through the synergistic interaction of CAM and SAM, thereby improving feature extraction efficiency and network representational capacity, ultimately optimizing model performance (Zeng and He, 2024, Lv and Su, 2024). To enhance the model’s performance and adaptability in potato miss-seeding detection under complex field backgrounds and high-dust environments during agricultural operations, this study incorporates an attention module into the final fusion output of three-scale branches, corresponding to the output of the CSP module. The features are subsequently passed directly to the detection head. The channel branch first computes two sets of statistics, with a length of C, using global average pooling (AvgPool) and global max pooling (MaxPool). These statistics are then mapped non-linearly using a shared two-layer perceptron and processed through a sigmoid function to obtain the channel weights. The input features are then re-weighted channel-wise to generate intermediate features. The spatial branch concatenates the averaged-pooled intermediate features and max-pooled intermediate features along the channel dimension, applies a 7×7 convolution, and processes the result through a sigmoid function to obtain spatial weights. These spatial weights are then element-wise multiplied with the intermediate features to produce the output features. To control model complexity, the channel compression ratio is set to 16. This modification does not alter the anchor box or post-processing configuration, nor does it affect the loss function or training strategy, facilitating direct reuse and deployment on embedded platforms.

Let the fused neck feature be F ∈*R*^(*C*×*H*×*W*). In the channel branch, global average and max pooling are used to extract statistics, which are then passed through a shared two-layer perceptron to obtain the channel weights as shown in Equation 6.


(6) $$M_{c}\left(F\right)=\sigma \left(MLP\left(AvgPool\left(F\right)\right)+MLP\left(MaxPool\left(F\right)\right)\right)$$

Where σ denotes the sigmoid function $M_{c}$ ∈*R*^(*C*×1×1), producing $F^{\prime }$ =· $M_{c}\left(F\right)$ ⊗ F . In the spatial branch, the intermediate feature is averaged-pooled and max-pooled along the channel dimension, concatenated, and convolved to generate the spatial weightsas shown in Equation 7.


(7) $$M_{s}\left(F^{\prime }\right)=\sigma \left(f^{\left(k\times k\right)}\left(\left[AvgPool_{c}\left(F^{\prime }\right);MaxPool_{c}\left(F^{\prime }\right)\right]\right)\right)$$

Where $M_{s}$ ∈*R*^(1×*H*×*W*), yielding the final output *F″* ·=· $M_{s}$ ( $F^{\prime }$ ) ⊗ $F^{\prime }$ . In this study, the module is inserted sequentially at the output of the CSP module, with a channel compression ratio *r* = 16 and a spatial convolution kernel size k = 7

The YOLOv5s object detection framework employs the weighted Non-Maximum Suppression (NMS) algorithm, with its calculation formula provided in Equation 8. This algorithm filters candidate bounding boxes based on Intersection over Union (IoU) through the following steps: firstly, all bounding boxes within the same category are sorted by confidence scores in descending order; subsequently, the bounding box with the highest score is selected; finally, the IoU between this box and the remaining boxes is calculated, and those exceeding a preset threshold are discarded. In potato miss-seeding detection, due to the similar shapes and sizes of potato seeds, the detection algorithm often generates multiple overlapping bounding boxes, resulting in increased IoU values. When applying weighted NMS, only one bounding box may be retained while other overlapping potato seed bounding boxes are erroneously excluded, leading to biased detection results.


(8) $$S_{i}=\{\begin{array}{c} S_{i},\;\;\;\;\;\;\;IoU\left(M,b_{i}\right)<N_{t}\\ 0,\;\;\;\;\;\;\;\;IoU\left(M,b_{i}\right)\geq N_{t} \end{array}$$

Where $S_{i}$ represents the score of the *i*-th detection box, M denotes the detection box with the highest score, $b_{i}$ corresponds to the *i*-th detection box, and $N_{t}$ is the preset IoU threshold.

The performance of NMS is highly sensitive to the selection of *N_t_*, an overly high threshold may lead to missed detection, while an excessively low threshold risks false positives. To address the loss of detection targets caused by improper threshold settings, this study adopts Soft-NMS to refine the original NMS algorithm, with its calculation formula detailed in Equation 9.


(9) $$S_{i}=\{\begin{array}{c} S_{i},\;IoU\left(M,b_{i}\right)<N_{t}\\ S_{i}\left(1-IoU\left(M,b_{i}\right)\right),\text{ I}oU\left(M,b_{i}\right)\;\geq N_{t} \end{array}$$

Soft-NMS enhances detection performance by progressively reducing the scores of overlapping detection boxes instead of directly discarding low-score boxes, offering a more flexible processing approach (Ma et al., 2024; Zhang et al., 2022).

### 4.4 Model training

#### 4.4.1 Test rig and model parameters

This experiment was conducted on an Ubuntu 16.04 operating system using PyCharm 1.13.0 as the development environment with Python 3.9. The computer configuration included an Intel Core i7–8700 CPU, NVIDIA GTX 3070Ti GPU, and 32 GB RAM. The model training parameters were set as follows: input image dimensions for potato miss-seeding detection were 2048×1536, the SGD optimizer was used for gradient optimization with a momentum of 0.9, an initial learning rate of 0.01 (maintained at 0.01 throughout training), weight decay of 0.0005, a batch size of 8, and 150 epochs of training.

#### 4.4.2 Indicators for model evaluation

To validate the effectiveness of the detection model, this study employs Precision (*P*), Recall (*R*), and mean Average Precision (*mAP*) as evaluation metrics, with their calculation formulas provided in Equations 10–12. Precision reflects the accuracy of predictions for both proper seed placement and miss-seeding status in the seed spoons. Recall measures the model’s ability to detect these two states, while *mAP* serves as a comprehensive metric for evaluating the model’s overall accuracy. The closer the above metric values are to 1, the better the detection performance of the model.


(10) $$P=\frac{TP}{TP+FP}$$

(11) $$R=\frac{TP}{TP+FN}$$

(12) $$mAP=\frac{1}{N}\sum_{i=1}^{N}AP_{i}$$

Where, TP (True Positive) represents the number of positive samples correctly identified by the model; FP (False Positive) denotes the number of negative samples incorrectly classified as positive; FN (False Negative) indicates the number of positive samples that the model failed to detect; mAP (Average Precision) is the area under the Precision-Recall (P-R) curve for each category; and N is the total number of detection categories.

#### 4.4.3 Ablation experiment

Based on the original YOLOv5s algorithm, the CBAM attention mechanism and improved non-maximum suppression (NMS) are respectively introduced. Ablation experiments numbered I, II, III, and IV are designed on a custom dataset, with the experimental results shown in **Table 1** Results of ablation tests.

**Table 1:** Results of ablation tests.

Test no.	Module setting		P/%	R/%	mAP/%
	CBAM	Soft_NMS			
I	–	–	96.02	96.31	99.12
II	✓	–	96.90	96.50	99.20
III	–	✓	98.30	96.10	99.20
IV	✓	✓	98.30	99.40	99.40

“√” indicates that the module is added; “-” indicates that the module is not added.

In Test I (original YOLOv5s), the precision (*P*), recall (*R*), and mean average precision (*mAP*) were 96.02%, 96.31%, and 99.12%, respectively, demonstrating high baseline performance. In Test II with the addition of CBAM, *P* increased by 0.88% to 96.90%, *R* improved by 0.19% to 96.50%, and *mAP* rose by 0.08% to 99.20%, indicating that CBAM enhances feature extraction capabilities and improves detection accuracy in complex backgrounds. In Test III using Soft-NMS, *P* significantly increased by 2.28% to 98.30%, *mAP* reached 99.20%, but *R* decreased by 0.21% to 96.10%, validating the advantage of Soft-NMS in reducing false positives while suggesting a potential weakening of recall capability for some samples. In Test IV combining CBAM and Soft-NMS, *P* reached 98.30%, *R* achieved 99.40%, and *mAP* attained 99.40%, respectively representing improvements of 2.28%, 3.09%, and 0.28% over Test I, demonstrating that the synergistic interaction of the two modules significantly optimizes model performance, particularly in minimizing missed and false detections.

#### 4.4.4 Test result display

**Figure 10:**
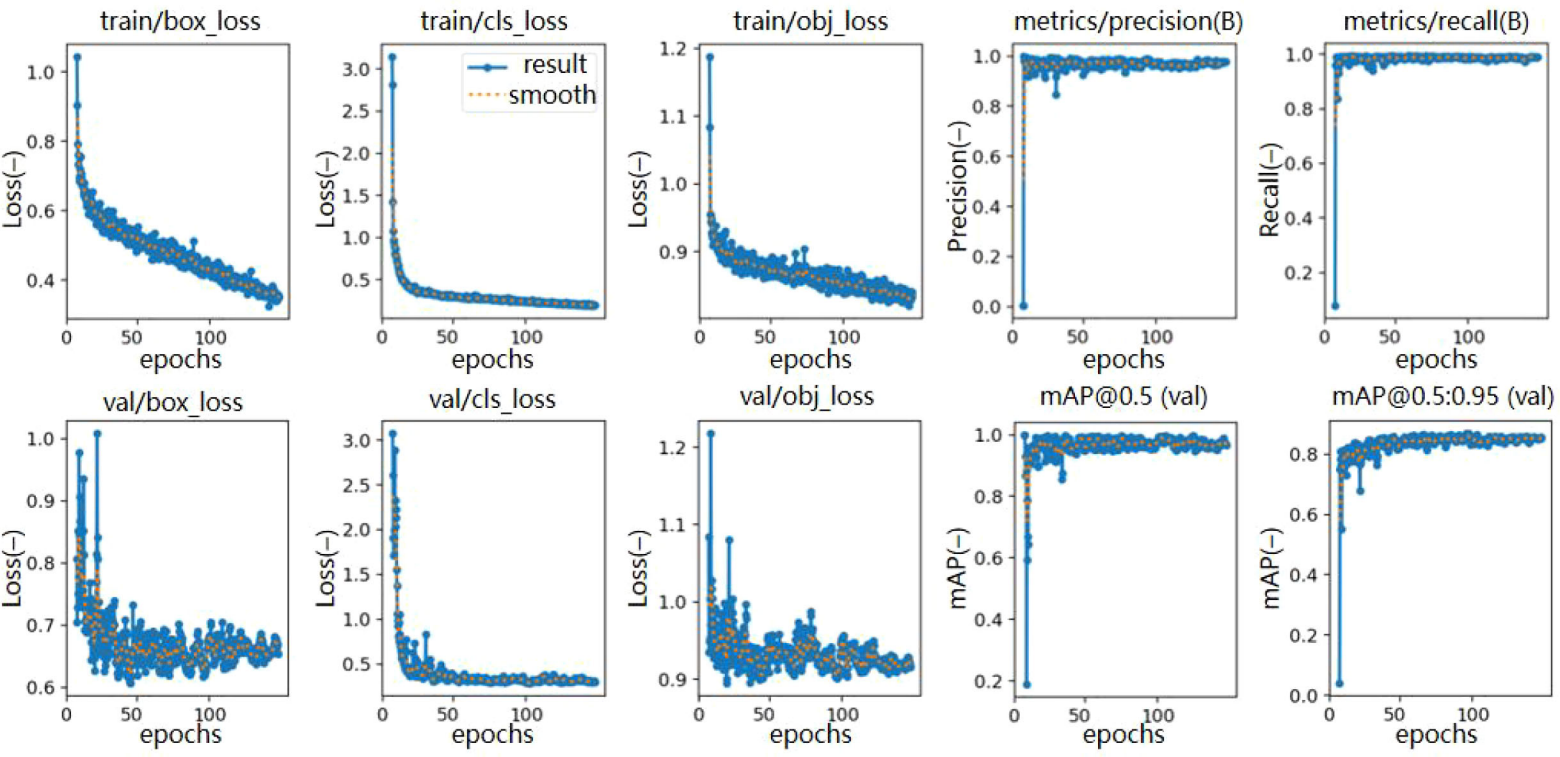
Loss and evaluation metric change charts of the improved model training. During the training of the improved detection model, the loss decreases with each epoch, and metrics such as precision and recall are good and stable, indicating that the model training is effective.

Analysis of the loss variation curve of the improved network reveals that the loss value decreases significantly within the first 50 epochs, gradually converges between 50 and 100 epochs, indicating that the proposed model achieves loss convergence with fewer training cycles. After 100 epochs, the network integrating the CBAM attention mechanism and the improved non-maximum suppression (Soft-NMS) demonstrates higher accuracy stability during training, approaching a stable value more closely compared to the original YOLOv5s algorithm, as shown in **Figure 10**.

Compared to existing methods, the improved YOLOv5s algorithm proposed in this study demonstrates more pronounced advantages in target feature extraction and overlapping target suppression. The traditional YOLOv5s framework is often affected by background interference such as soil, shadows, and weeds in complex field environments, leading to insufficient feature representation and, consequently, limited detection accuracy. Moreover, the standard Non-Maximum Suppression (NMS) method it relies on often causes missed detections due to “hard rejection” when processing highly overlapping potato seed bounding boxes, negatively impacting recall rate. To address these issues, this study introduces the CBAM attention mechanism and the Soft-NMS algorithm into YOLOv5s. The CBAM (Convolutional Block Attention Module) enhances the expression of key features through channel and spatial attention weighting, significantly improving robustness in complex backgrounds. The Soft-NMS algorithm softens the suppression strategy, reducing false positives while mitigating missed detections, which greatly improves the recognition ability of overlapping targets. Ablation experiments further validate the effectiveness of these improvements. After integrating CBAM and Soft-NMS, the model demonstrates substantial improvements in precision, recall rate, and mean Average Precision (*mAP*). These results indicate that the improved YOLOv5s model offers superior performance and adaptability in agricultural miss-seeding detection tasks compared to existing approaches.

## 5 Performance tests

### 5.1 Test condition

**Figure 11:**
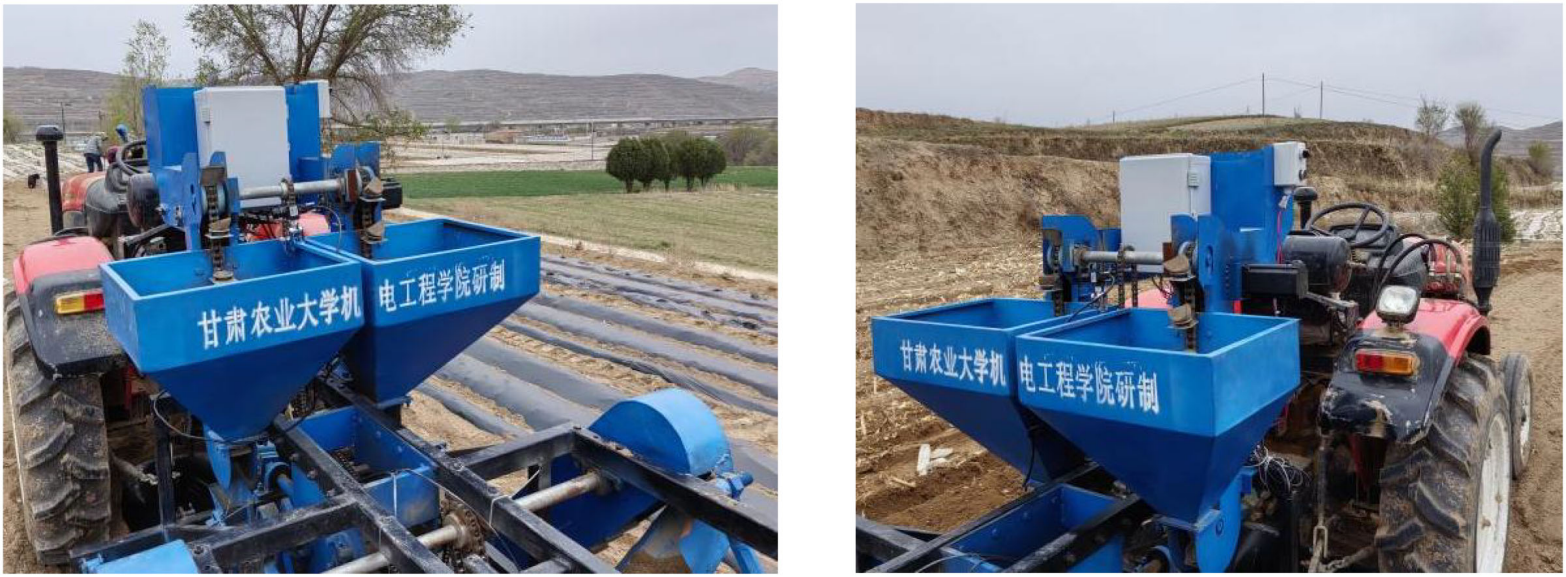
Field testing diagram. The diagram illustrates the field testing process after the installation and debugging of the miss-seeding, automatic compensation, and control system.

Building upon the aforementioned principles, systematic hardware and software designs were implemented, completing the manufacturing of the potato planter. The miss-seeding detection, automatic compensation, and control systems were installed and debugged. Powered by tractor traction, experimental validation confirmed the system’s feasibility and reliability, with the testing process illustrated in **Figure 11**.

### 5.2 Test content and indicators

#### 5.2.1 Reseeding system test

The primary objective of this experiment was to simulate scenarios where the reseeding belt lacked potato seeds, testing whether the preparatory seed preparation system could automatically replenish seeds and whether the reseeding system could effectively reseed. A total of five batches were conducted, with each batch controlled to have 50 miss-seeding instances; thus, the theoretical number of preparatory seeds per batch was also 50. **Table 2** Reseeding system test results presents the experimental results of the reseeding system. The data indicate that while the preparatory seed reseeding success rate was high, there were 1 and 2 unsuccessful cases in batches 3 and 4, respectively, caused by mechanical jamming. The preparatory seed reseeding success rate was calculated as the number of reseeding success divided by the actual number of preparatory seeds. The reseeding success rate of preparatory seeds in **Table 2** demonstrate high performance (no less than 85% across trials), with failures primarily attributed to potato seed bouncing or collisions during the compensation process, which significantly altered the trajectory of the potato seeds, leading to unsuccessful reseeding.

**Table 2:** Reseeding system test results.

Test batch	Preparatory seeds (theoretical)	Preparatory seeds (actual)	Preparatory seeds success rate	Number of reseeding success	Reseeding success rate
1	50	50	100%	45	90%
2	50	50	100%	44	88%
3	50	49	98%	42	86%
4	50	48	96%	41	85%
5	50	50	100%	43	86%

#### 5.2.2 Performance test of miss-seeding detection and reseeding system

The miss-seeding detection and reseeding system must not compromise the operational quality of the seed-metering device, so its performance is evaluated based on the reseeding success rate under varying linear speeds of the seed-metering chain. In this experiment, the planting spacing was fixed at 110 mm using cut potato seeds, with seed-metering chain linear speeds set to 0.2 m/s, 0.3 m/s, and 0.4 m/s, representing low-speed, medium-speed, and high-speed operating conditions, respectively. Each speed level was tested three times, with a theoretical seed count of 300 potatoes per trial. The objective was to validate the reliability of the improved YOLOv5s-based detection system, preparatory seed preparation system, and reseeding mechanism under dynamic conditions. To ensure accurate data collection, the experiment recorded the original seeding count, original miss-seeding count, reseeding count, final seeding count, and final theoretical seeding count, with their relationships defined as shown in Equation 13.


(13) $$\left\{ \begin{array}{l} n_{4}=n_{0}+n_{1}\\ n_{3}=n_{4}-n_{1}+n_{2} \end{array}\right.$$

*n*_o_ represents the original seeding count (unit: grain), which is the actual number of seeds sown by seed-metering device without any compensatory measures; *n*_1_ represents the original miss-seeding count (unit: grain), which is the number of seeds missed due to seed pickup failure during the seeding process; *n*_2_ represents the reseeding count (unit: grain), which is the additional number of seeds placed by the reseeding mechanism after miss-seeding detection; *n*_3_ represents the final seeding count (unit: grain), which equals the sum of the original seeding and reseeding numbers, reflecting the actual final seeding result in the field; *n*_4_ represents final theoretical seeding count (unit: grain), which equals the sum of the original seeding and the original miss-seeding numbers, representing the total number of seeds that should be sown under ideal conditions without any missed seeding. This value is used as a theoretical benchmark to evaluate the seeding accuracy of the system.

Based on statistical data, the original miss-seeding rate ( $\eta_{1}$ ) could be calculated as the ratio of miss-seeding count to the theoretical seeding count without the system as shown in Equation 14.


(14) $$\eta_{1}=\frac{n_{1}}{n_{4}}\times 100\%$$

The final miss-seeding rate ( $\eta_{2}$ ) after using the miss-seeding detection and reseeding system was calculated as the final miss-seeding count divided by the theoretical seeding count as shown in Equation 15.


(15) $$\eta_{2}=\frac{n_{1}-n_{2}}{n_{4}}\times 100\%$$

The reseeding success rate ( $\eta_{3}$ ) of the system is shown in Equation 16.


(16) $$\eta_{3}=\frac{n_{2}}{n_{1}}\times 100\%$$

By recording the seed-taking process of the seed-metering device, the miss-seeding detection process, and the reseeding process during each trial group, statistical analysis was conducted for each trial group, and the results are presented in **Table 3** Results of seed taking and reseeding performance tests.

**Table 3:** Results of seed taking and reseeding performance tests.

Seeding speed	Test number	*n*_0_	*n*_1_	*n*_2_	$\eta_{1}$	$\eta_{2}$	$\eta_{3}$
0.2m/s	1	272	17	14	5.88%	1.04%	82.35%
	2	266	15	13	5.34%	0.71%	86.67%
	3	269	15	12	5.28%	1.06%	80.00%
	Averages	269	15	13	5.28%	0.70%	86.67%
0.3m/s	1	270	19	16	6.57%	1.04%	84.21%
	2	269	17	14	5.94%	1.05%	82.35%
	3	273	21	18	7.14%	1.02%	85.71%
	Averages	270	19	16	6.57%	1.04%	84.21%
0.4m/s	1	265	27	22	9.25%	1.71%	81.48%
	2	274	30	24	9.87%	1.97%	80.00%
	3	271	29	23	9.67%	2.00%	79.31%
	Averages	270	28	23	9.40%	1.68%	82.14%

Based on the data in **Table 3** Results of seed taking and reseeding performance tests, the performance of the improved YOLOv5s-based spoon-chain potato seed-metering device’s miss-seeding detection and scraper-belt compensation system exhibits distinct patterns under different sprocket linear speeds. At a low speed of 0.2 m/s, the original miss-seeding rate remained within 5.28%–5.88% across three repeated trials, averaging 5.28%, indicating a low probability of missed seeding. With the intervention of the reseeding system, the final miss-seeding rate fluctuated between 0.70% and 1.04%, averaging 0.70%, while the reseeding success rate stabilized at 80.00%–86.67%, averaging 86.67%. This demonstrates that the improved YOLOv5s model efficiently identifies miss-seeding positions at low speeds with minimal false positives or negatives, and the scraper-belt compensation mechanism achieves high operational precision, ensuring stable detection and reseeding performance under low-speed conditions.

When the sprocket linear speed increased to 0.3 m/s, the system exhibited slight performance variations. The original miss-seeding rate rose moderately to 6.57%–7.14% (average: 6.57%), and the final miss-seeding rate increased slightly to 1.02%-1.05% (average: 1.04%), yet remained low. The reseeding success rate ranged from 82.35% to 85.71% (average: 84.21%), showing a minor decline compared to low-speed conditions but maintaining high effectiveness. This confirms that the improved YOLOv5s model retains strong detection capabilities at medium speeds, and the compensation mechanism remains stable.

However, at a high speed of 0.4 m/s, system performance degraded significantly. The original miss-seeding rate surged to 9.25%–9.87% (average: 9.40%), aligning with findings from (Zhang, 2024), where faster speeds reduce seed population refill efficiency between adjacent holes in the seed-filling zone, increasing miss-seeding rate. The final miss-seeding rate rose to 1.71%–2.00% (average: 1.68%), reflecting amplified mechanical vibrations and dynamic instability in the seed-metering device under high-speed operation, which complicates detection. The reseeding success rate declined to 79.31%–81.48% (average: 82.14%), indicating delays in the dynamic response of detection and compensation components under high-speed conditions, impairing reseeding accuracy.

Performance trends across speeds reveal the system’s strengths and limitations. At low speed (0.2 m/s), the integration of the improved YOLOv5s model with CBAM, Soft-NMS, and the scraper-belt mechanism achieves near-ideal balance between detection precision and reseeding efficiency, making it highly suitable for precision planting scenarios with low speed requirements. Medium-speed (0.3 m/s) results confirm the system’s viability under typical field conditions, with only minor performance degradation. However, the increased miss-seeding rate and reduced reseeding success at high speed (0.4 m/s) highlight current design limitations under dynamic stress, necessitating further optimization for high-speed applications.

## 6 Conclusion

This study developed an improved YOLOv5s-based spoon-chain potato seed-metering device miss-seeding detection and reseeding system, integrating a preparatory seed scraper-belt mechanism to achieve precise miss-seeding identification and rapid reseeding. By incorporating the CBAM attention mechanism and Soft-NMS, the model’s detection accuracy was enhanced, with ablation experiments demonstrating precision, recall, and *mAP* of 98.30%, 99.40%, and 99.40%, respectively. The system operates in real time on the NVIDIA Jetson TX2 platform, meeting embedded application requirements. Tests showed that at low speed (0.2 m/s), the original miss-seeding rate was 5.28%, reduced to 0.70% after reseeding, with a reseeding success rate of 86.67%; at medium speed (0.3 m/s), the original miss-seeding rate was 6.57%, reduced to 1.04%, with a success rate of 84.21%; at high speed (0.4 m/s), the original miss-seeding rate was 9.40%, reduced to 1.68%, with a success rate of 82.14%. The system performed excellently at low-to-medium speeds but slightly declined at high speeds due to vibrations. The scraper-belt mechanism achieved a preparatory seed preparation success rate exceeding 96%,which effectively improved the continuity and stability of the operation.

The experimental results not only validated the system’s performance across varying speed conditions but also uncovered several critical patterns. The system maintained high stability and precision at low and medium speeds, demonstrating the strong robustness of the improved algorithm in complex environments. However, performance deteriorated under high speeds, highlighting that the interplay between detection–compensation response time and vibration suppression capacity constituted a major limiting factor. These findings further demonstrates that the sequential collaborative design of visual detection and mechanical compensation is feasible and effective in agricultural scenarios, though improvements remained necessary under extreme operating conditions.

Despite the significant progress achieved, several limitations persist. Performance decline during high-speed operations indicated that both the real-time responsiveness of detection–compensation and the dynamic stability of the mechanical structure under intense vibrations require further enhancement. In addition, the method primarily relied on visual sensors, which were susceptible to interference from changes in lighting, dust occlusion, and motion blur, potentially affecting detection reliability in extreme field environments. The system’s decision-making solely relies on visual information, without fusion feedback from multimodal sensors such as infrared and pressure sensors, which limits its robustness and adaptability across different environments. Future research will focus on lightweight optimization and heterogeneous acceleration of detection algorithms to reduce inference latency and enhance responsiveness under high speeds. At the same time, a multi-sensor fusion strategy will be introduced to enhance the system’s perception redundancy and reliability. Furthermore, the system’s generalizability will be further validated in different crop sowing scenarios, and, when combined with intelligent operational planning in precision agriculture, its application potential and value for broader deployment will be significantly expanded.

## Data Availability

The original contributions presented in the study are included in the article/supplementary material. Further inquiries can be directed to the corresponding author.
